# Protein-x of hepatitis B virus in interaction with CCAAT/enhancer-binding protein α (C/EBPα) - an *in silico *analysis approach

**DOI:** 10.1186/1742-4682-8-41

**Published:** 2011-10-28

**Authors:** Ashraf Mohamadkhani, Parisa Shahnazari, Zarrin Minuchehr, Armin Madadkar-Sobhani, Mahmoud Jeddi Tehrani, Ferdous Rastgar Jazii, Hossein Poustchi

**Affiliations:** 1 Digestive Disease Research Centre, Tehran University of Medical Sciences, Tehran, Iran; 2 Monoclonal Antibody Research Centre, Avicenna Research Institute, ACECR, Tehran, Iran; 3 National Institute of Genetic Engineering and Biotechnology, NIGEB, Tehran, Iran; 4 Department of Bioinformatics, Institute of Biochemistry and Biophysics (IBB), University of Tehran, Tehran, Iran; 5 Life Sciences Department, Barcelona Supercomputing Centre (BSC), 08034 Barcelona, Spain

**Keywords:** Protein-x, Hepatitis B, Tertiary structure prediction

## Abstract

**Background:**

Even though many functions of protein-x from the Hepatitis B virus (HBV) have been revealed, the nature of protein-x is yet unknown. This protein is well-known for its transactivation activity through interaction with several cellular transcription factors, it is also known as an oncogene. In this work, we have presented computational approaches to design a model to show the structure of protein-x and its respective binding sites associated with the CCAAT/enhancer-binding protein α (C/EBPα). C/EBPα belongs to the bZip family of transcription factors, which activates transcription of several genes through its binding sites in liver and fat cells. The C/EBPα has been shown to bind and modulate enhancer I and the enhancer II/core promoter of HBV. In this study using the bioinformatics tools we tried to present a reliable model for the protein-x interaction with C/EBPα.

**Results:**

The amino acid sequence of protein-x was extracted from UniProt [UniProt:Q80IU5] and the x-ray crystal structure of the partial CCAAT-enhancer α [PDB:1NWQ] was retrieved from the Protein Data Bank (PDB). Similarity search for protein-x was carried out by psi-blast and bl2seq using NCBI [GenBank: BAC65106.1] and Local Meta-Threading-Server (LOMETS) was used as a threading server for determining the maximum tertiary structure similarities. Advanced MODELLER was implemented to design a comparative model, however, due to the lack of a suitable template, Quark was used for *ab initio *tertiary structure prediction.

The PDB-blast search indicated a maximum of 23% sequence identity and 33% similarity with crystal structure of the porcine reproductive and respiratory syndrome virus leader protease Nsp1α [PDB:3IFU]. This meant that protein-x does not have a suitable template to predict its tertiary structure using comparative modeling tools, therefore we used QUARK as an *ab initio *3D prediction approach. Docking results from the *ab initio *tertiary structure of protein-x and crystal structure of the C/EBPα- DNA region [PDB:1NWQ] illustrated the protein-binding site interactions. Indeed, the N-terminal part of 1NWQ has a high affinity for certain regions in protein-x (e.g. from Ala76 to Ser101 and Thr105 to Glu125).

**Conclusion:**

In this study, we predicted the structure of protein-x of HBV in interaction with C/EBPα. The docking results showed that protein-x has an interaction synergy with C/EBPα. However, despite previous experimental data, protein-x was found to interact with DNA. This can lead to a better understanding of the function of protein-x and may provide an opportunity to use it as a therapeutic target.

## Background

Human beings are the natural hosts for the hepatitis B virus (HBV), which infects approximately 350 million individuals worldwide each year. The 3.2-kb long viral genome is partially double stranded and contains four identified open reading frames (ORFs). The ORFx encodes the 154 amino acids of protein-x with a molecular mass of 17.5 kDa, which has not been found in mature virions and is not accompanied by a nucleocapsid particle [1,2]. The hepatocarcinogenesis of the hepatitis B virus in association with protein-x has been studied further in recent years [3]. However, the 3D structure of protein-x is currently unknown, and hence specific functions of this protein are not well understood. Protein-x has no counterparts in any of its hosts and is conserved among mammalian hepadnavirus [1]. Although the mechanism by which this protein mediates hepatocellular carcinogenesis is not yet understood, it is known that it is a multifunctional regulator that transactivates viral and host genes through a variety of promoters [4]. Researchers have shown that this protein is not a DNA binding molecule, and that it is therefore, not a typical transactivator [5]. Most promoters which are activated by protein-x attach to the transcription factors belonging to the basic leucine zipper (bZIP) family. The In vitro interaction assay and electrophoretic mobility shift assays have shown that protein-x increases the DNA binding activity of the CCAAT/enhancer-binding protein α (C/EBPα) through direct interaction with the enhancer [6-9]. The C/EBPα is expressed mainly in highly differentiated cells such as liver and fat cells [10,11]. Domain analysis of protein-x indicates that the central region (amino acids 78-103) is necessary for a direct interaction with C/EBPα [12]. However, the complete form of protein-x is necessary for the synergistic activation of the HBV pregenomic promoter which suggests that the interaction of protein-x with C/EBPα enhances the transcription of the HBV pregenomic promoter, leading to the effective life cycle of HBV in hepatocytes [7,12].

Experimentally, protein-x has defied x-ray crystallography and nuclear magnetic resonance [5]. Since no 3D structure of the protein is available, determination of the secondary structure and tertiary structures of protein-x can be regarded as an interesting area of research in order to elucidate its function [13]. Here, we have sought to define a structural model for protein-x and describe its interaction with the CCAAT/enhancer-binding protein α (C/EBPα) using computational methods. The prediction of the tertiary structure of protein-x could have valuable applications, such as the possibility of controlling the cellular transactivating function and the development of hepatocellular carcinoma. This can also have an impact on the HBV life cycle in hepatocytes induced via this protein. The protein as a whole may act as an excellent target for designing specific drugs to treat HBV infection.

## Methods

### Data sets

The sequences in this study were retrieved from the public databases: National Center for Biotechnology Information (NCBI), http://www.ncbi.nlm.nih.gov/[14]; EBI (The European Bioinformatics Institute) http://www.ebi.ac.uk/[15] and UniProt/ExPASy (Swiss Bioinformatics Resource) http://expasy.org/tools/[16]. The protein-x [UniProt:P0C681] and its deduced amino acid sequence was retrieved from the UniProt database and the x-ray crystallography of the partial CCAAT-enhancer α [PDB:1NWQ] was retrieved from The Protein Data bank (PDB) http://www.rcsb.org/pdb[17].

### Similarity and multiple alignments

HBx [PDB:P0C681] was used to conduct similarity search against the UniProt data base [18] using blastp algorithm. Protein sequences were aligned using Clustal-X [19] and Jalview [20], and analyzed using GeneDoc http://www.nrbsc.org/gfx/genedoc/[21]. LOMETS, threading results were used for identifying similarity between protein-x and the crystallized structure PDB.

### Amino acid analysis

MEGA software was used for the amino acid sequence analysis, this software has been developed to use molecular biology data for estimating evolutionary distances, reconstructing phylogenetic trees and computing basic statistical quantities, such as nucleotide and amino acid frequencies, transition/transversion bases, codon frequencies (codon usage tables), and the number of variable sites in specified segments of the amino acid sequences [22]. PROSITE http://prosite.expasy.org/ was searched for protein-x motif contents [23] and the DiANNA 1.1 web server was used to predict the disulfide bond topology, the server is available at http://cassandra.dsi.unifi.it[24].

### Tertiary structure modeling and Validation

The LOMETS is an on-line web service for protein structure prediction [25], it generates tertiary structures by collecting high-scoring target-to-template alignments from 8 locally-installed threading programs (FUGUE, HHsearch, MUSTER, PPA, PROSPECT2, SAM-T02, SPARKS, SP3). LOMETS can easily find the best 10 threading models selected from 160 models by the confidence score. It also selects the top 10 target-template alignments of individual threading servers and full-length models built by MODELLER http://cassandra.dsi.unifi.it[26].

Modeller as a restrained-based modeling structure begins with an alignment of the sequence to be modeled (target) with a related known 3D structure (template). In this study, the align2d function of the MODELLER program was used to align protein-x sequence with the sequence of the porcine reproductive and respiratory syndrome virus leader protease Nsp1α [PDB:3IFU] as template (the target-template alignment was used to build the model by satisfaction of spatial restraints). The initial model was then refined (eight times) using the loop-refine program. QUARK was used for as *ab initio *folding and structure prediction of small proteins that predicted the 3D model only from amino acid sequences http://zhanglab.ccmb.med.umich.edu/QUARK/. The NIH's Laboratory for Structural Genomics and Proteomics procheck [27] and verify-3D [26] was carried out to evaluate the tertiary structure predicted. Protein structure illustrations were generated with the PyMOL Molecular Graphics Software [28].

### Docking

The binding sites of the C/EBPα that interact with protein-x were docked using several docking software's and the results were compared with each other. The docking servers included the Z-dock which has implemented the fast Fourier transform to search and evaluate all possible binding modes based on shape complementary, de-solvation energy and electrostatics http://zdock.bu.edu/; the PatchDock Molecular docking algorithm was based on shape complementary principles to evaluate suitable binding positions using FireDock [29], Hex http://www.loria.fr/~ritchied/hex_server/ and the RosettaDock protein-protein docking server which predicts the structure of protein complexes with respect to the structures of individual components and an approximate binding orientation [30].

## Results and Discussion

### Predicted structural properties

A homology search of protein-x using several protein sequence databases from ExPASy-blast, displayed a large conserved domain which was found in hepadnaviruses, representing a non-homologous protein. In order to understand the physico-chemical properties of protein-x, the frequency of amino acids in protein-x were determined using the MEGA software. The isoelectric point of protein-x in this calculation was 8.45 and the percentage of basic residues was higher than that of the acidic ones. A good example of this was the global propensity **(GP_ai_^1^) **which was calculated using the following formula: **GP_ai_^1 ^= Pω^ai^/P_ai_^1^**, where **Pω^ai ^**is the percentage of individual amino acids in the protein, and Pai^1 ^is the percentage of individual amino acids obtained from UniProtKB/Swiss-Prot (ExPASy), data released on 31-May-11 was used. The global propensities of Arg and Ser in protein-x were 1.53 and 1.49 respectively (global propensity > 1.2 shows a significant abundance of the related amino acid). The total charge of protein-x was positive, which could be due to the presence of high amounts of Arg in this protein. The high percentage of Ser also shows the highly phosphorylated form of Ser. Moreover, this analysis showed that the percentages of amino acids such as Arg, Cys, Leu and Phe were relatively high and those of Tyr, Thr, Ile, Gln and Asn were low in contrast.

PROSITE (release 20.48) motifs revealed that protein-x included functional sites for protein kinase C and casein kinase II associated phosphorylation, and N-myristoylation.

The predicted disulfide bonds include 7-17, 7-26, 7-61, 7-69, 7-115 and 115-148, regions, which were obtained by the DiANNA web server.

### Threading analysis and model construction

LOMETS generates full-length 3D protein structural predictions, therefore, an initial 3D model was generated for protein-x, which was selected for further refinement. The threading results in this model were applied to the PPA-I program (PPA-I, is a simple sequence profile-profile alignment approach combined with secondary structure matches). The data showed that the crystal structure of the porcine reproductive and respiratory syndrome virus leader protease Nsp1α [PDB:3IFU] was the best template with a z-score = 6.287, coverage = 0.961, 23% identity. Moreover, the TM-score which was calculated using the Zang lab server was 0.1677 [31]. A TM-score > 0.5 indicates a model of correct topology and a TM-score < 0.17 means a random similarity, therefore we had to refine the model and in order to predict a better model, the model was applied as a template using MODELLER for the purpose of refinement (loop refined). Validation of protein-x was checked by the Ramachandran plot in the PROCHECK server to improve the refinement factors resulting in the absence of amino acids in the disallowed region and the presence of one residue in the generously allowed regions. The TM-score and RMSD between the final and initial models were 0.12 and 2.26, respectively. Moreover, the protein backbone may probably move away from the native structure.

**Figure 1:**
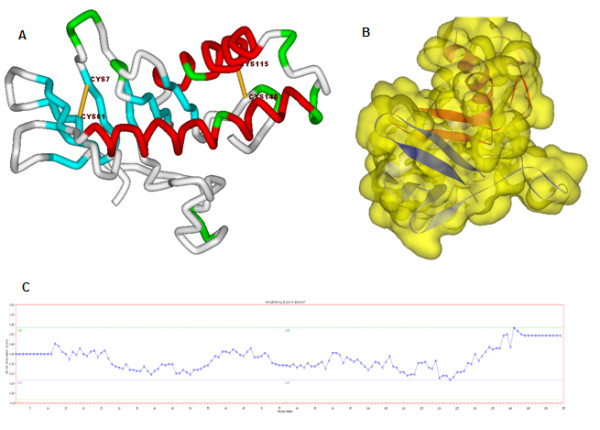
**Tertiary structure of protein-x using QUARK as ab initio model**. A: The location of the disulfide bond in this model is Cys7-Cys61 and Cys115-Cys148. B: Tertiary structure of protein-X C: The verify-3D plot of protein-x, as can be seen the dots are upper than the threshold.

Indeed, because of the low similarity between the templates and protein-x, comparative models were not successful. To find a good model, we used QUARK, a computer algorithm for *ab initio *protein folding and protein structure prediction. Protein-x is a relatively small protein, therefore prediction of the tertiary structure using QUARK was possible, since QUARK constructs the correct 3D protein model from amino acid sequence. For this reason, the results were evaluated using Verify-3D, which analyzes the compatibility of an atomic model (3D) with its own amino acid sequences (1D). The first model was chosen as the best model due to the evaluation mentioned above. Each residue was assigned to a structural class based on its location and environment (α, β, loop, polar, non-polar, etc.). A collection of good structures were used as a reference to obtain a score for each of the 20 amino acids in this structural class. The *ab initio *tertiary structure of protein-x derived from QUARK and the verifid-3D plot are shown in Figure 1, where it can be clearly observed that, there are two disulfide bonds; Cys7-Cys61 and Cys115-Cys148. This data is compatible with the disulfide predictions obtained from the DIANNA by prediction tools.

### Docking of C/EBPα to protein-X

According to previous studies regarding the role of protein-x in binding the C/EBPα DNA, the C/EBPα-DNA complex [PDB:1NWQ] was used for comparative purposes. The *ab initio *structures of protein-x and crystal structure of the C/EBPα-DNA complex [PDB:1NWQ] were used to calculate docking using different docking servers, such as Patchdock, Firedoc, Rosettadock, Zdock and Hex. The best model was chosen from the overlapped results.

**Figure 2:**
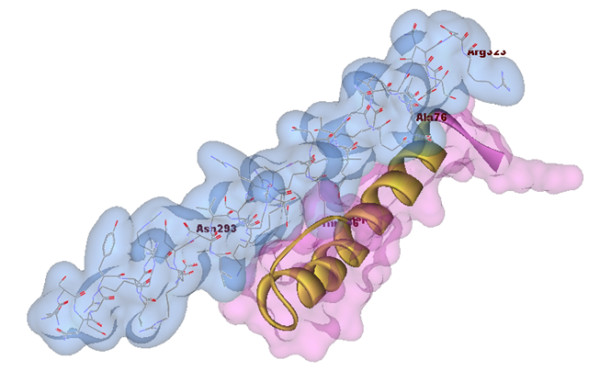
**The FireDock results**. The light blue surface is a part of C/EBPα (Asn281-Arg 323), the light pink surface is protein-x from Ala66 to Arg76.

**Figure 3:**
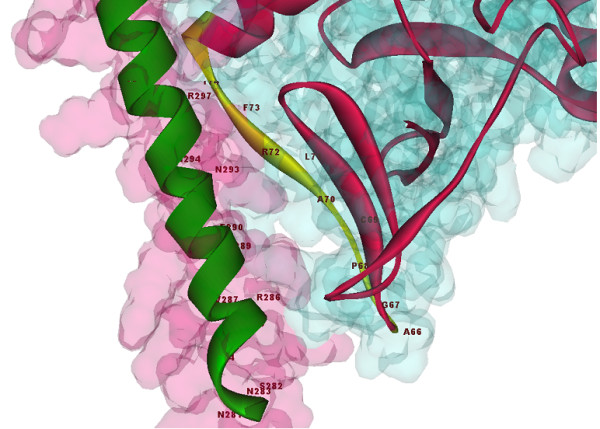
**The Binding site region between protein-x and C/EBPα**. Docking result using RosettaDock; Ala66 - Ala76 in protein-x are bonded to C/EBPα (283-301).

**Figure 4:**
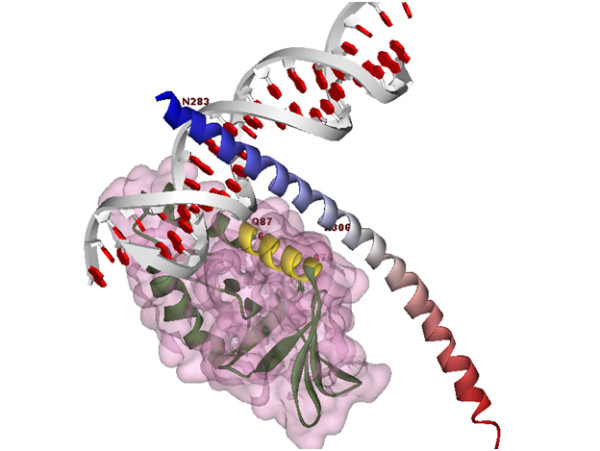
**A complex of protein-x, C/EBPα-DNA Domain and DNA molecule**. The surface region is protein-x, The PatchDock result shows that protein-x from Ala76 to Ser101 are bonded to C/EBPα. DNA molecule has interactions with the N-terminal region of C/EBPα-DNA Domain from Gln87 to Ser101 in protein-x.

In all docking results, C/EBPα was found to contact protein-x at the Asn281-Asn307 position (N-terminal of the C/EBPα-DNA complex domain) (Figure 2). The Rosettadock results showed that the residues from Ser65 to Ala76 of protein-x are in close proximity to C/EBPα (Figure 3). Other docking data showed that residues from Thr105 to Glu125 of protein-x were involved with the C/EBPα-DNA complex domain; this does not mean that all residues in this regions, have interactions with C/EBPα. A vivid illustration of these interactions is demonstrated by Asp114 from protein-x and the Arg 288 of C/EBPα. A docking result using Patchdock showed interactions between C/EBPα DNA and protein-x (Figure 4). Indeed, the N-terminal of the C/EBPα-DNA binding region is exposed to certain regions in protein-x. In other words, the region spanning Asn281-Asn307 in C/EBPα could be a good candidate for epitope prediction with respect to vaccine preparation.

An interesting point about the results of this study is the DNA interactions with protein-x, according to Figure 4, protein-x has interactions with C/EBPα in the region covering Ala76 to Ser101. Data from this study revealed that the C-terminal part of protein-x has an important role in the direct interaction with the b-Zip domain of C/EBPα, which is in agreement with experimental data [1,12].

## Conclusion

Even though, many studies have been carried out on the pathogenesis of protein-x in the hepatitis B virus over the last decade, the tertiary structure of this protein still remains obscure. Protein-x is expressed in hepatocytes, and acts as part of a protein-protein complex to enhance the transcriptional efficacy of b-zip proteins and to alter their DNA binding specificities [7]. This protein serves as a conserved domain and cannot be organized into an evolutionary classification.

Furthermore, protein analysis showed that protein-x possesses a positive charge because of its propensity for residues such as Arg. One hypothesis for the protein-x action is the increased calcium release in the cytoplasm [5]. The phosphorylation of HBx induces the activation of calcium protein kinase and consequently, stimulates protein-x calcium signaling effects. Protein-x has several predicted Ser and Thr phosphorylation sites, which are compatible with the experimental work for phosphorylation when expressed both in insect and HepG2 cells [32,33]. Docking results showed that the N-terminal of the C/EBPα-DNA domain (from 281 to 323) are involved in protein-x. These findings reveal a new perspective in drug design using an appropriate linear epitope which can inhibit the function of protein-x.

In conclusion, this 3D model may provide some insights into the hierarchical structure of protein-x, leading to a better understanding of the function of this protein and its interaction with the cellular proteins, which can lead to the development of new treatments.

## List of abbreviations

C/EBPα: (CCAAT/enhancer-binding protein α); bZIP: (basic leucine zipper); HBV: (hepatitis B virus).

## Competing interests

The authors declare that they have no competing interests.

## Authors' contributions

All authors contributed both to the research and the discussion and they have read and approved the final manuscript.
